# Osteosarcomatous Divergence in Dedifferentiated Liposarcoma Presenting as a Colonic Mass

**DOI:** 10.1155/2019/8025103

**Published:** 2019-07-15

**Authors:** Jeenal Gordhandas, Grace Lin, Ann M. P. Tipps, Somaye Y. Zare

**Affiliations:** Department of Pathology, University of California San Diego, La Jolla, San Diego, CA, USA

## Abstract

Dedifferentiated liposarcomas most commonly arise in the retroperitoneum, accounting for 10% of liposarcomas. Heterologous differentiation occurs in 5-10% of dedifferentiated liposarcomas; however, divergent osteosarcomatous differentiation is rare. We report a rare case of initial presentation of dedifferentiated liposarcoma with osteosarcomatous component as a colonic mass in a 72-year-old man. The tumor is mainly composed of bony trabeculae with intervening highly atypical cells and adjacent high-grade mesenchymal nonlipogenic tumor, as well as areas of well-differentiated liposarcoma. Immunohistochemical studies showed diffuse positivity for SATB2 in the atypical cells and fluorescence in situ hybridization revealed high-level amplification of MDM2 gene, supporting the diagnosis of well-differentiated and dedifferentiated liposarcoma with heterologous osteosarcomatous differentiation.

## 1. Introduction

Liposarcomas are the most common malignant mesenchymal neoplasms, accounting for about 20% of all sarcomas [1]. Dedifferentiated liposarcoma (DDL) refers to relatively abrupt transition to a nonlipogenic sarcoma of variable histologic grade arising in primary or recurrent atypical lipomatous tumor (ALT)/well-differentiated liposarcoma (WDL) [2]. Progression to DDL occurs in up to 10% of WDL, most commonly in the retroperitoneal region, and is associated with increased risk of local recurrence and metastasis. The WDL/DDL group is characterized by amplification of genes located at chromosomal region 12q 13-15, particularly MDM2 gene [3]. Dedifferentiated areas most frequently resemble an undifferentiated pleomorphic sarcoma, whereas heterologous differentiation occurs in about 5-10% of the DDLs [2]. Myogenic differentiation is the most common type of heterologous differentiation; however, other mesenchymal lineages including osteosarcomatous, chondrosarcomatous, and angiosarcomatous are also seen, albeit rarely. Herein, we report an unusual initial presentation of a DDL with osteosarcomatous components as a colonic mass causing compression of the colon and constipation.

## 2. Case Report

**Figure 1:**
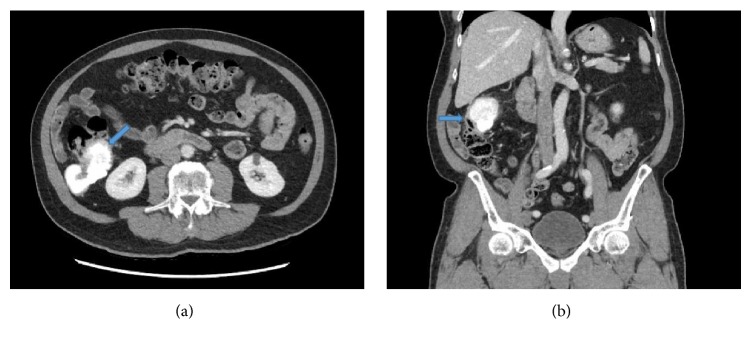
Contrast-enhanced CT scan images of right pericolonic mass: (a) axial and (b) coronal images demonstrate large partly exophytic lobulated mass (blue arrow) arising from the mid ascending colon with heterogeneously enhancement, consistent with mineralization or calcifications.

A 72-year-old male presented with a history of weakness and constipation, for which he underwent colonoscopy, revealing a submucosal mass in the right colon, compressing on the lumen extrinsically. The mucosa overlying the mass was intact and colonoscopy showed an adjacent 12 mm polyp, which turned out to be a tubular adenoma. He had history of removal of previous tubular adenomas on prior colonoscopies. Computed tomography (CT) imaging showed a 7.0 cm lobulated mass arising from mid-ascending colon with exophytic growth and foci of calcifications. CT findings were consistent with a mesenchymal tumor and carcinoma was less likely. No fatty component was reported (Figure 1). Subsequently, the patient underwent laparoscopic right hemicolectomy and tumor was resected.

**Figure 2:**
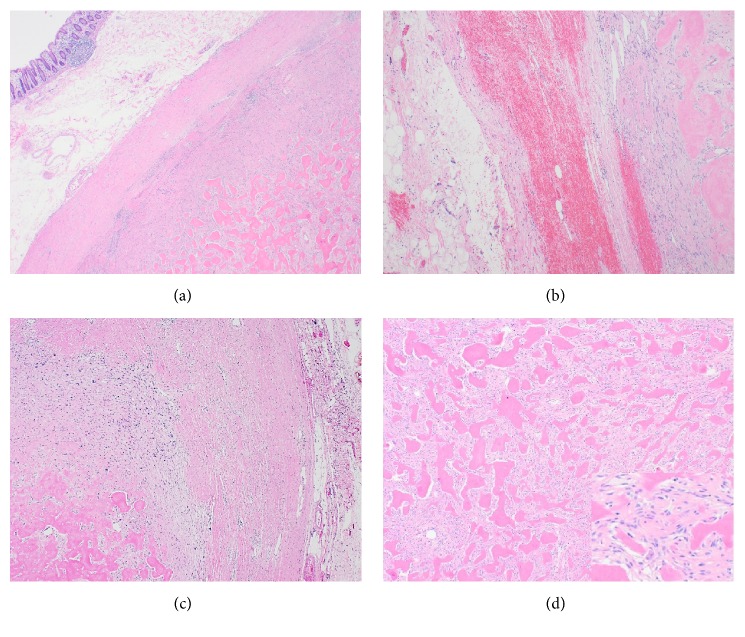
(a) A low power image showing dedifferentiated liposarcoma with osseous differentiation involving the colonic wall (20X). (b) Dedifferentiated liposarcoma with osseous differentiation adjacent to well-differentiated liposarcoma (left) (20X). (c) Dedifferentiated liposarcoma with osseous differentiation and highly atypical cells (40X). (d) Areas of low-grade osteogenic differentiation with well-formed bone trabeculae and intervening spindle cells (40X). The insert shows high power image of the spindle cells with mild atypia (100X).

**Figure 3:**
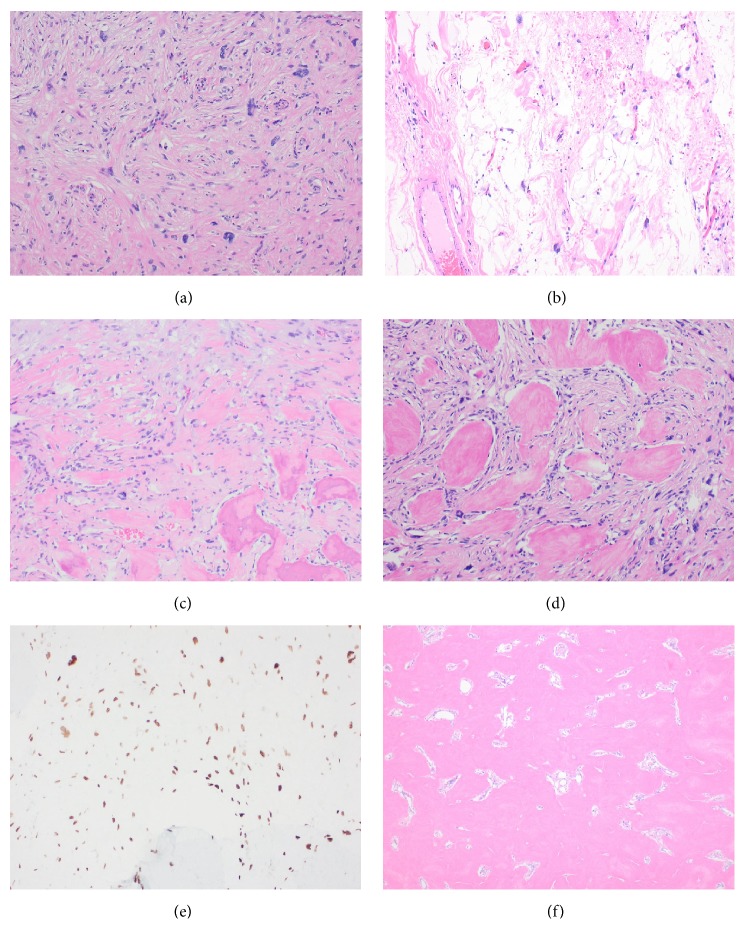
(a) Cellular high-grade nonlipogenic sarcoma component composed of spindle and pleomorphic tumor cells (100X). (b) Focal transition into a well-differentiated liposarcoma with atypical stromal cells (100X). (c, d) Nonlipogenic sarcoma with osteoid matrix and highly atypical cells, resembling a high-grade osteosarcoma (100X). (e) Immunohistochemical staining for SATB2 is positive in osteoblastic cells and intervening atypical cells. (f) Areas of thick interconnected trabeculae of immature woven bone lacking osteoblastic rimming (40X).

Gross examination of the right hemicolectomy specimen demonstrated a firm and calcified intramural mass in the posterior wall of ascending colon, extending into the mesocolic fat, without involvement of the mucosa. Microscopically, distinct histological patterns were identified, with the majority of the tumor being composed of a central ossified core surrounded by a minor component of a high-grade mesenchymal nonlipogenic tumor and adipose tissue. The central bone forming component showed areas of osteoid matrix with highly atypical cells resembling a high-grade osteosarcoma as well as areas of more mature appearing woven bone trabeculae with intervening mildly atypical spindle cells similar to a low-grade osteosarcoma (LGOS). A thin rim of a high-grade sarcoma composed of atypical spindled and pleomorphic cells was identified in the periphery of the bony mass. In addition, small foci of mature-appearing adipose tissue containing rare atypical hyperchromatic stromal cells were detected adjacent to the high-grade sarcoma and at the retroperitoneal resection margin, raising consideration of a WDL (Figures 2 and 3). The findings suggested that WDL may have arisen in the retroperitoneum and secondarily involved the colon with a DDL component with heterologous osseous differentiation.

On immunohistochemical (IHC) studies, SATB2 was diffusely positive in the atypical cells between bony trabeculae and in high-grade sarcomatous areas without evident bone formation. High and low molecular weight keratin (pancytokeratin), CD117, DOG-1, desmin, SMA, and S-100 stains were negative. Fluorescence in situ hybridization (FISH) revealed high-level amplification of MDM2-gene, supporting the diagnosis of retroperitoneal WDL with DDL showing heterologous osteosarcomatous differentiation and forming a mass involving the ascending colon.

## 3. Discussion

The term dedifferentiated liposarcoma (DDL) was first introduced by Evans in 1979 to define the morphological progression from a well-differentiated liposarcoma to a nonlipogenic sarcoma. DDL commonly occurs in the retroperitoneum showing a variable histological appearance, most frequently a high-grade sarcoma which is much less aggressive than other types of high‐grade pleomorphic sarcomas [2]. A minority of cases show divergent differentiation, usually with myogenic and less commonly angiosarcomatous and/or osteochondromatous components [4]. Different patterns of osseous differentiation in DDL have been reported. Osseous tissue can show severe atypia and resemble high-grade osteosarcoma, demonstrate bone trabecula with only mildly atypical cells simulating low-grade osteosarcoma, or, most commonly, resemble heterotopic bone [5]. Yamashita et al. reported the bone tissue formed in DDLPS is mainly neoplastic, regardless of its morphology and maturity, and is a result of osteogenic differentiation of the tumor cells [5]. Our case demonstrated both areas of low-grade and high-grade osteogenic differentiation.

Presentation of a heavily ossified deep visceral mass is an unusual finding, which proposes a differential diagnosis of heterologous osseous differentiation in a mesenchymal or epithelial neoplasm or rarely extraskeletal osteosarcoma. In our case, the initial presentation of the patient with constipation, colon mass, and concurrent and previous tubular adenomas raised a clinical concern for colorectal carcinoma. Imaging findings, however, were consistent with a mesenchymal tumor of the colon with no fatty component. When present, imaging findings of a fatty component could be helpful in diagnosis of a liposarcoma. Although rare, osseous differentiation in soft tissue sarcomas and colorectal carcinomas have been reported [6–8]. In the current case, histologic findings and immunohistochemical studies excluded carcinoma, leiomyosarcoma, gastrointestinal stromal tumor, and nerve sheath tumors. Albeit extremely rare, a case of extraskeletal osteosarcoma (ESOS) of the colon has been reported [9]. Although MDM2 status was not alluded to in the aforementioned case report, widespread sampling did not identify well-differentiated liposarcoma in any of the sections [9]. In our case, the presence of surrounding, subtle WDL led us to consider a diagnosis of DDL with heterologous differentiation. This diagnosis was further supported by high-level amplification of MDM2 gene on FISH studies. With WDL component extending to, and likely beyond, the retroperitoneal margin, we concluded the neoplasm may have arisen in the retroperitoneum and secondarily involved the colon with a DDL component and heterologous osteosarcomatous differentiation.

Evaluation of MDM2 and CDK4 expressions by IHC and FISH have been increasingly used for distinction of WDL/DDL from other sarcomas [3]. However, MDM2 amplification is not limited to WDL/DDL and has been observed in LGOS [10], including cases of LGOS with dedifferentiation which contain areas resembling pleomorphic sarcoma or high-grade osteosarcoma [11]. In addition, Yamashita et al. reported a series of ESOS with MDM2 amplification in which background features suggestive of ALT/WDL and thus DDL were not identified [12]. Therefore, the detection of MDM2 amplification in the osteosarcomatous part is not sufficient to distinguish between ESOS and DDL with heterologous osteosarcomatous differentiation. In our case, a combination of the tumor location, histologic findings, MDM2 results, and most importantly recognition of the WDL component supported the diagnosis of WDL/DDL. In diagnostic work-up for cases of bone-forming soft tissue sarcomas with MDM2 amplification, it should be noted that frequency of DDL is much higher compared to ESOS; therefore, the possibility of a DDL should be carefully evaluated; thus a thorough sampling of the fatty tissue surrounding the tumor becomes imperative.

In summary, DDL with osteosarcomatous dedifferentiation is a rare entity. Our case illustrates an unusual initial presentation of a DDL with osteosarcomatous components as a colonic mass. The microscopic recognition of WDL was the key finding in diagnosis of this case.
